# Alleviation of mercury toxicity to a marine copepod under multigenerational exposure by ocean acidification

**DOI:** 10.1038/s41598-017-00423-1

**Published:** 2017-03-23

**Authors:** Yan Li, Wen-Xiong Wang, Minghua Wang

**Affiliations:** 1 Center for Marine Environmental Chemistry and Toxicology, College of the Environment & Ecology, Xiamen University, Xiamen, 361102 China; 2 Key Laboratory of the Ministry of Education for Coastal and Wetland Ecosystems, Xiamen University, Xiamen, 361102 China; 3 Division of Life Science, The Hong Kong University of Science and Technology (HKUST), Clearwater Bay, Kowloon Hong Kong

## Abstract

Ocean acidification (OA) may potentially modify the responses of aquatic organisms to other environmental stressors including metals. In this study, we investigated the effects of near-future OA (pCO_2_ 1000 μatm) and mercury (Hg) on the development and reproduction of marine copepod *Tigriopus japonicus* under multigenerational life-cycle exposure. Metal accumulation as well as seven life history traits (survival rate, sex ratio, developmental time from nauplius to copepodite, developmental time from nauplius to adult, number of clutches, number of nauplii/clutch and fecundity) was quantified for each generation. Hg exposure alone evidently suppressed the number of nauplii/clutch, whereas single OA exposure negligibly affected the seven traits of copepods. However, OA exposure significantly alleviated the Hg inhibitory effects on number of nauplii/clutch and fecundity, which could be explained by the reduced Hg accumulation under OA. Such combined exposure also significantly shortened the development time. Thus, in contrast to earlier findings for other toxic metals, this study demonstrated that OA potentially mitigated the Hg toxicity to some important life traits in marine copepods during multigenerational exposure.

## Introduction

Ocean acidification (OA) caused by absorption of increasing anthropogenic CO_2_, with a continuous decline in pH^1^ is now widely regarded as a major threat to global marine biodiversity. The atmospheric CO_2_ increased steadily from a preindustrial level (~280 μatm) to a contemporary concentration with about 400 μatm^2, 3^. Average ocean surface pH has dropped by 0.1 units (a 26% increase in the hydrogen ion concentration) since the industrial revolution^4–6^. It is predicted that the atmospheric pCO_2_ will break the barrier of 1000 μatm by the end of 2100, resulting in a decrease in seawater surface pH of 0.3–0.5 units (pH 7.6–7.9)^7^. Increased seawater pCO_2_ can result in hypercapnia and acidosis^8^ which may cause re-allocation of energy into growth and reproduction due to mobilization of energy costly acid-base regulatory processes to fight against internal pH reduction. Accordingly, OA has been shown to perturb a range of physiological processes including calcification^9^, survival^10^, fertilization^11^, embryonic development^12^, metabolism^13^, and reproduction^14^ in calcifying and non-calcifying organisms.

In addition to the increase in global atmospheric CO_2_ levels, anthropogenic activities also significantly promote the mercury (Hg) emission to the atmosphere^15^, which will finally enter into marine environments^16, 17^. Thus, OA and Hg pollution may co-occur in marine environments. Indeed, Hg pollution has been a serious environmental concern for marine environments in China^18–20^, which contributed approximately 28% to the global Hg emissions in the atmosphere. For example, the maximum level of total Hg (T-Hg) was reported to be 2.7 µg/L in the seawater in Jinzhou Bay, about three orders of magnitude higher than the background level^20^. Hg toxicity is often ascribed to its high affinity for the SH groups in endogenous biomolecules including proteins and enzymes, hence resulting in their dysfunctions (e.g., oxidative damage) and subsequently producing multi-toxicities in the organisms^21, 22^. To our knowledge, only one study examined the impacts of elevated pCO_2_ (i.e., 380, 850 and 1500 µatm with equal pH values of 8.10, 7.85 and 7.60, respectively) on Hg accumulation in the early stages of the squid *Loligo vulgari* ^23^. The results demonstrated that, in the whole egg strand and paralarvae, OA enhanced Hg uptake efficiency with the maximum level at the 850 µatm, but for the embryo Hg displayed a minimum concentration factor under the 850 µatm treatment. Thus, OA might result in a joint effect of pH/protons on the binding efficiency of biological surfaces and/or interference with physiological processes in the organisms, leading to change in Hg accumulation. Nevertheless, no previous study has investigated the combined effects to marine organisms produced by OA and Hg, let alone the long-term multigenerational impacts.

Recently, several studies have focused on the impact of OA and metals such as cadmium (Cd) and copper (Cu) on marine animals^24–26^. OA is expected to modify the bioavailability of metals^27^. For example, the toxic free-ion concentration of Cu increased by as much as 115% in coastal waters in the next 100 years due to reduced pH^28, 29^, and may lead to increased Cu toxicity to marine copepod *Amphiascoides atopus* ^28^ and polychaete *Arenicola marina* ^24^ under OA exposure. Similarly, Ivanina *et al*. reported that OA exacerbated the negative effects of Cd on immunity in marine bivalves *Crassostrea virginica* and *Mercenaria mercenaria* ^30^, despite the fact that free Cd ion decreased or remained unchanged due to reduced pH caused by OA^23, 28^. Conversely, the bioaccumulation of some metals decreased while others increased in the eggs of the cuttlefish *Sepia officinalis* and in the early stages of the squid *Loligo vulgari* under increased pCO_2_^23, 31^. For Hg, OA does not affect its speciation in seawater, since this metal forms strong complexes with chloride, the concentration of which will not change by decreasing pH in seawater^27^. However, OA may influence Hg toxicity to marine organisms by altering the physiological processes and/or metal accumulation in the biota.

In the present work, we used the harpacticoid copepod *Tigriopus japonicus* as a model species given its ease of culture, rapid life cycle and pedigree in ecotoxicological studies including the OA impacting assessments^32–35^. This copepod inhabits tide pools on rocky shores along the coasts in the Western Pacific including Japan, South Korea, and China^36^, and thus it may have suffered from multi-stresses (e.g., OA and Hg pollution) due to human activities. We specifically examined the combined effects of OA and Hg on the multigenerational life history of this copepod. Earlier studies were mainly devoted to short-term effects (e.g., single generation effect) of OA or metal pollution, with very few on the long-term multigenerational exposure^35, 37^. In this study, *T. japonicus* were cultured for four consecutive generations (F0-F3) under the exposure of OA (1000 μatm) and Hg (at a nominal concentration of 1.0 µg/L) stress (alone or combined). Seven important life history traits, i.e., survival rate, sex ratio (F/M), developmental time from nauplius to copepodite, developmental time from nauplius to adult, number of clutches, number of nauplii/clutch and fecundity, as well as Hg accumulation, were measured for each generation.

## Results

### Hg accumulation in the copepods

**Figure 1:**
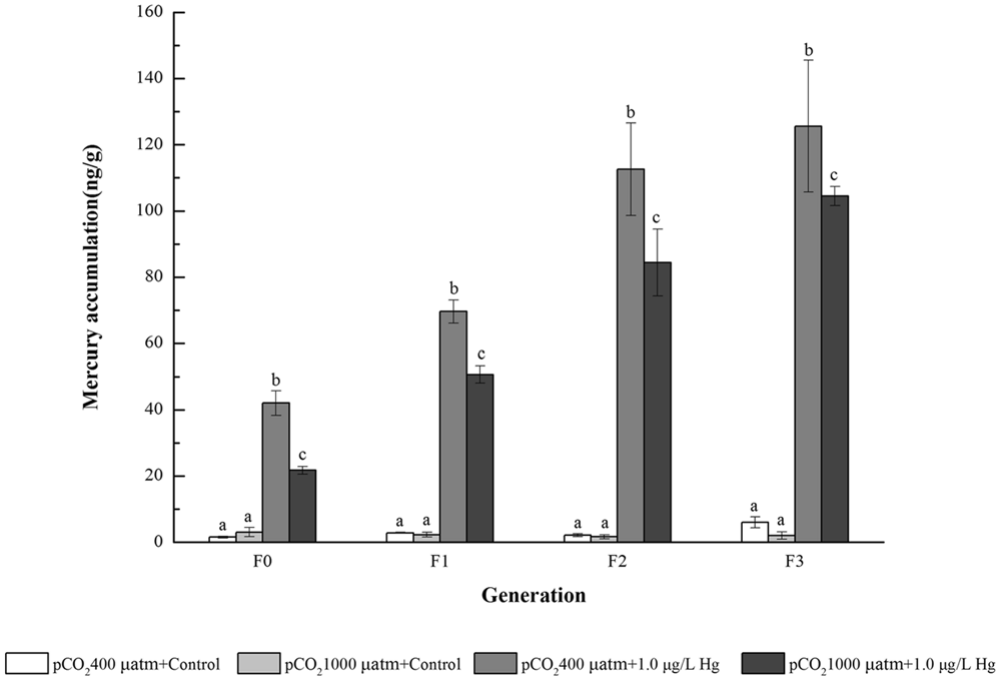
Total Hg contents in the adult copepod *Tigriopus japonicus* under multigenerational exposure to pCO_2_ and Hg. Data are described as means ± standard deviation (n = 3). Different letters indicate a significant difference among different treatments at *p* < 0.05.

In contrast to the ambient condition, both single Hg and OA plus Hg exposures significantly enhanced the Hg accumulation in the copepods at each generation, with a general tendency for higher Hg accumulation from F0 to F3 (Fig. 1). Compared with Hg treatment alone, the combined OA and Hg exposure decreased the T-Hg concentrations by 52, 73, 75, and 83%, respectively, for F0, F1, F2, and F3. These results strongly suggested that CO_2_ acidified seawater reduced the Hg accumulation in the copepods during multigenerational exposure. In addition, the dry-weight concentration factors (DCFs) were 42.1, 69.6, 112.6, and 125.6 L/kg, respectively, for F0, F1, F2, and F3 under Hg treatment alone, as compared to 21.8, 50.7, 84.4, and 104.5 L/kg for the combined OA plus Hg exposure.

### Survival rate, sex ratio (F/M), development time from nauplius to copepodite, and development time from nauplius to adult

**Figure 2:**
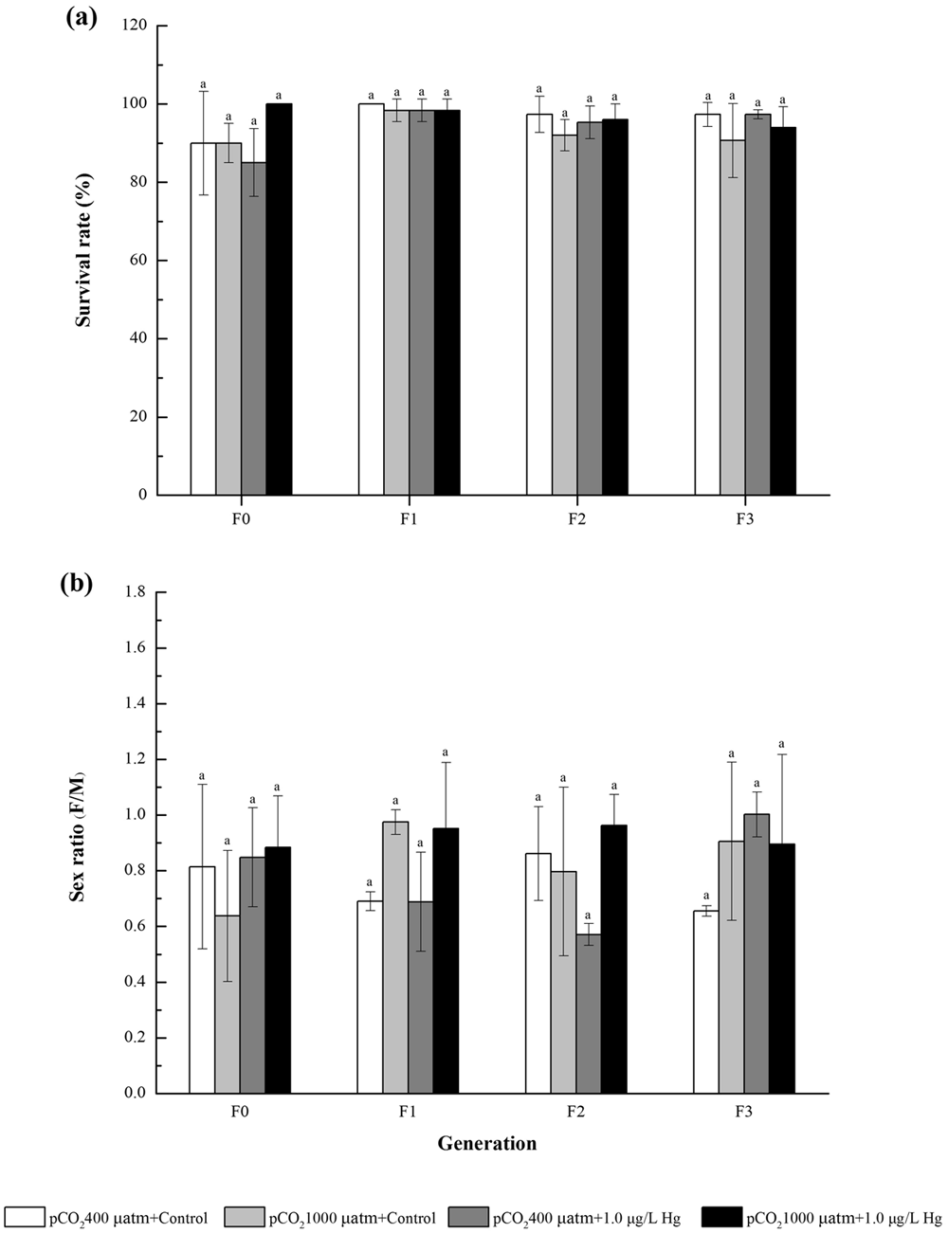
Effects of pCO_2_ and Hg on (**a**) survival rate and (**b**) sex ratio (F/M) in *Tigriopus japonicus* under multigenerational exposure. Data are described as means ± standard deviation (n = 3). Different letters indicate a significant difference among different treatments at *p* < 0.05.

**Figure 3:**
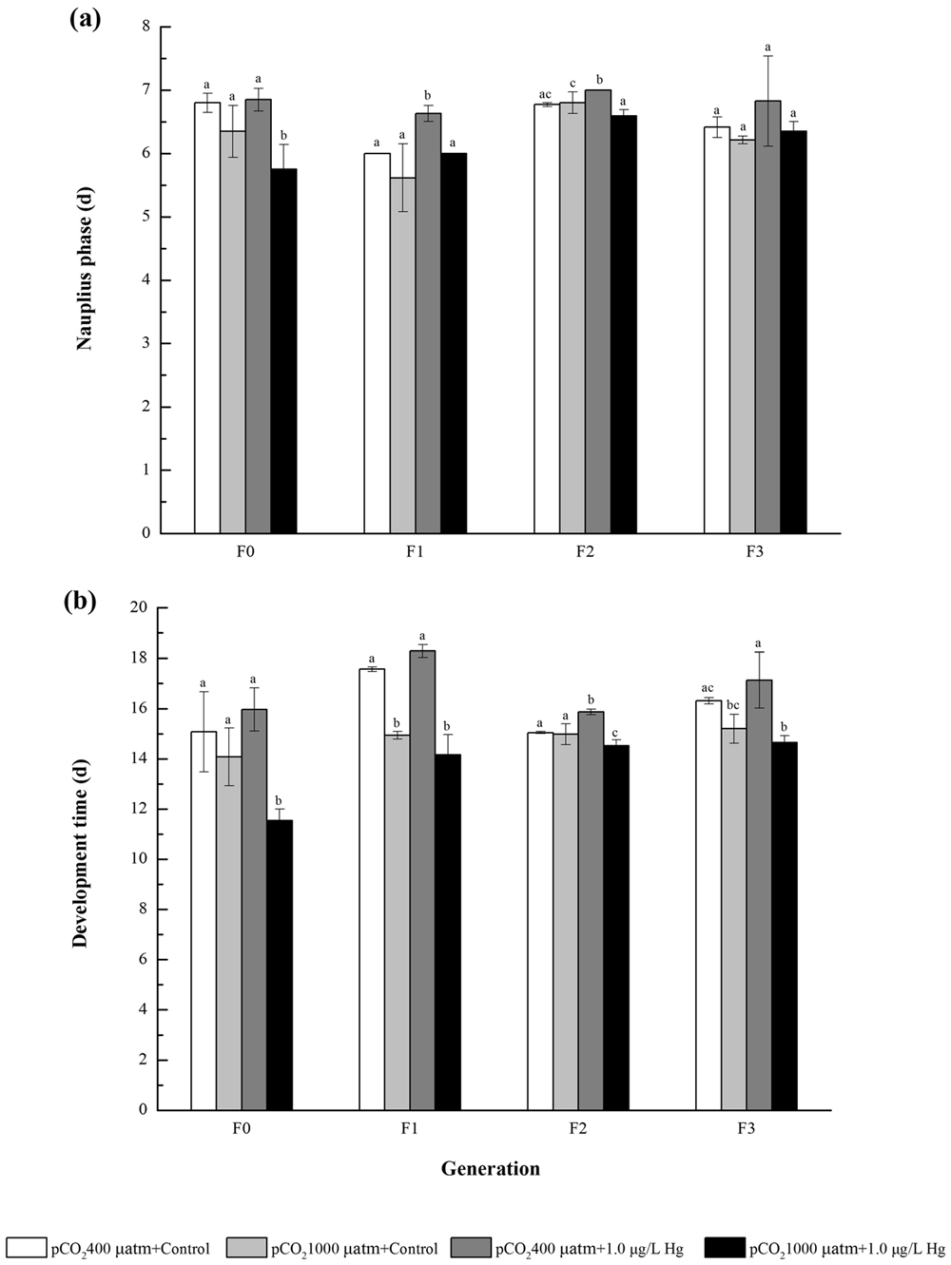
Effects of pCO_2_ and Hg on (**a**) nauplius phase (nauplius to copepodite) and (**b**) development time (nauplius to adult) in *Tigriopus japonicus* under multigenerational exposure. Data are described as means ± standard deviation (n = 3). Different letters indicate a significant difference among different treatments at *p* < 0.05.

Compared with the ambient condition, different OA and Hg treatments (alone or combined) exerted negligible impacts on survival rate and sex ratio during multigenerational exposure (Fig. 2). Additionally, OA exposure alone did not significantly affect development time from nauplius to copepodite (Fig. 3a) and development time from nauplius to adult (Fig. 3b) in most cases when compared with the ambient condition. However, the single Hg exposure trended to prolong these two life traits for F0-F3, although insignificant difference was observed under some circumstances. In combination, developmental time from nauplius to adult was significantly shortened, although development time from nauplius to copepodite showed little difference with the ambient condition under most circumstances.

### Number of clutches, number of nauplii/clutch, and fecundity

**Figure 4:**
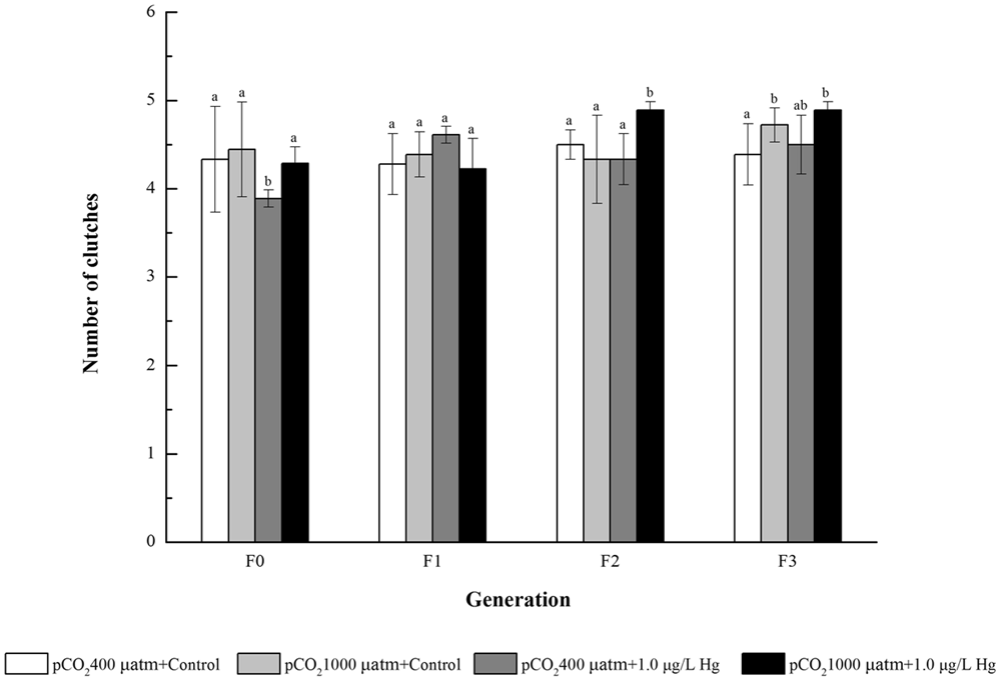
Effects of pCO_2_ and Hg on number of clutches in four generations of *Tigriopus japonicus* under multigenerational exposure. Data are described as means ± standard deviation (n = 3). Different letters indicate a significant difference among different treatments at *p* < 0.05.

**Figure 5:**
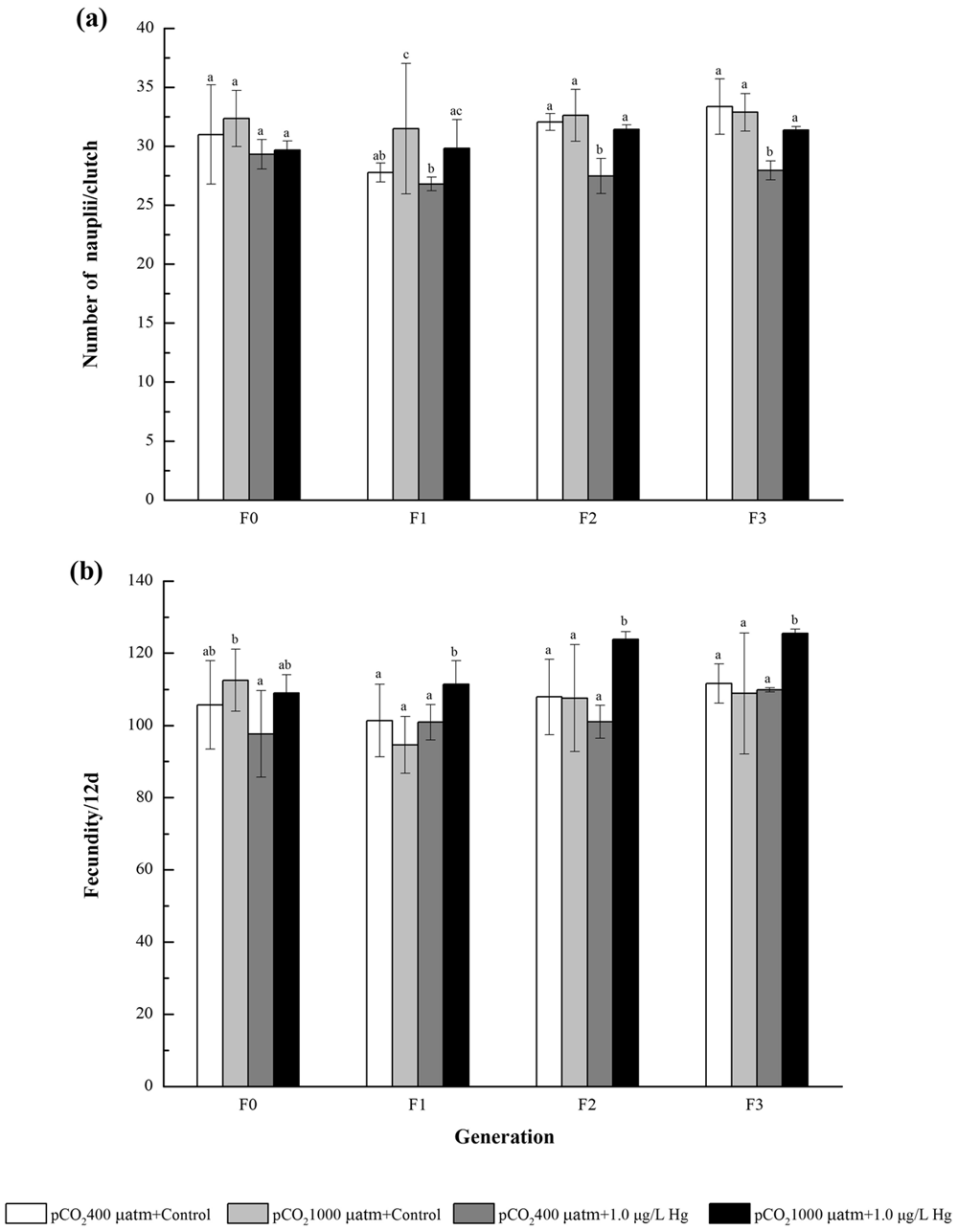
Effects of pCO_2_ and Hg on (**a**) number of nauplii/clutch and (**b**) fecundity/12 d in *Tigriopus japonicus* under multigenerational exposure. Data are described as means ± standard deviation (n = 3). Different letters indicate a significant difference among different treatments at *p* < 0.05.

In contrast to the ambient condition, number of clutches in most cases was not affected by OA or Hg pollution alone, but significantly increased in F2-F3 under the combined OA + Hg exposure (Fig. 4). OA alone negligibly affected the number of nauplii per clutch, whereas the single Hg exposure significantly inhibited the number of nauplii per clutch at later generations. Interestingly, the combined exposure did not significantly impact the number of nauplii per clutch during F0-F3 (Fig. 5a). OA or Hg exposure alone exhibited insignificant effect on fecundity at each generation, but the combined exposure strikingly increased the fecundity in the copepod of F1-F3 (Fig. 5b).

### Significant interaction between OA and Hg pollution

There was a significant interaction between OA and Hg in affecting the development time from nauplius to copepodite, development time from nauplius to adult, number of nauplii per clutch, and fecundity during multigenerational exposure (Table 1). Under combination, development time from nauplius to copepodite was significantly reduced in F0-F3 by contrast to the single Hg treatment, so did the development time from nauplius to adult. For instance, development time from nauplius to adult under single Hg exposure was 16.0, 18.3, 15.9 and 17.1 d for F0-F3, and they were shortened to 11.5, 14.2, 14.5 and 14.6 d, respectively, by the OA plus Hg exposure. Compared with the single Hg treatment, the combined exposure significantly increased the number of nauplii/clutch by 1.01, 1.11, 1.14 and 1.12 times for F0-F3. Similarly, total fecundity under the combined exposure was enhanced by 1.12, 1.10, 1.23 and 1.14 times, respectively, for F0-F3 by comparison with the single Hg exposure.

**Table 1:** The statistical difference of nauplius phase (nauplius to copepodite), development time (nauplius to adult), number of nauplii/clutch, fecundity/12 d between single Hg treatment and the combined OA plus Hg exposure during F0-F3 via a student’s t-test of two independent-samples (*p* < 0.05).

Life history traits	Sample 1: sample 2	Mean	t	df	*p*	95% Confidence Interval of the Difference	
						Lower	Upper
Nauplius phase	Single Hg exposure: OA plus Hg exposure	6.827:6.174	4.377	21.754	**<0.001**	0.343366	0.962424
Development time	Single Hg exposure: OA plus Hg exposure	16.812:13.719	5.849	21.53	**<0.001**	1.995245	4.191536
Number of nauplii/clutch	Single Hg exposure: OA plus Hg exposure	27.884:30.613	−5.498	140.85	**<0.001**	−3.710558	−1.747776
Fecundity	Single Hg exposure: OA plus Hg exposure	102.347:117.861	−7.347	142	**<0.001**	−19.68842	−11.33935

## Discussion

As expected, copepods significantly accumulated Hg under Hg exposure. The calculated DCFs of Hg in the copepods were 42.1–125.6 L/kg under the single Hg exposure, which was 1–2 orders of magnitude lower than those in other copepods measured in earlier works^38, 39^, highlighting the species-specificity for metal bioaccumulation. Additionally, lower Hg accumulation in our study can be explained by different durations of exposure, since the calculated DCFs may also incorporate metal sorption onto the copepod’s body, and this part may be released during the long-term multigenerational exposure. Moreover, Hg concentrations in the copepods trended to increase with increasing generations. For example, under Hg exposure alone, the Hg content in F3 increased by roughly 3 times when compared with that in F0. The enhanced accumulating tendency with generations could be attributed to maternal transfer of metals during multigenerational exposure^35, 40, 41^. Alternatively, an increased trend for Hg accumulation may be partially related to metallothionein (MT) induction in the copepods, since the maternally exposed animals would prepare to produce more MT to supply more binding sites for the internal metals during mutigenerational exposure^41^. It should be noted that the treated T-Hg contents in this work were comparable with Hg concentrations in several marine copepods in the environment^42–44^, and thus were environmentally relevant.

The most interesting finding in the present work was that OA significantly reduced Hg accumulation in copepods at each generation. There are several explanations for the decreased Hg accumulation under OA. First, the increased available H^+^ at OA may possibly compete with Hg to bind with the biotic ligands on biological membrane^45–47^. Such competition may result in less Hg internalization due to cationic competition. Previous studies also showed that cationic competition could contribute to reduced metal toxicity^48, 49^. Specifically, De Schamphelaere and Janssen performed a standard 30 d assay to investigate the effects of pH (5.5–7.5) on the chronic toxicity of zinc to juvenile rainbow trout *Oncorhynchus mykiss*, and found that enhanced H^+^ concentrations decreased the chronic zinc toxicity in fish by 2 times, suggesting a competitive effect between free zinc ions and hydrogen ions^48^. Alternatively, the lower pH would facilitate the protonation of phospholipid head groups, and the reduced charge could subsequently produce tighter packing of the phospholipids, resulting in a lower membrane permeability and diffusion for the metal complex within the membrane. Acidification of the external solution potentially displayed a negative effect on the passive diffusion and uptake of metals into cells. The aforementioned hypothesis was supported by an earlier work that the decrease from pH 7.0 to 5.5 prohibited the uptake of two lipophilic metal complexes Cd(diethyl-dithiocarbamate)_2_^0^ and Cd(ethyl-xanthate)_2_^0^ by freshwater algae, and lower metal accumulation was mainly caused by less membrane permeability due to the interaction of protons with phospholipids in the algal membrane^50^. Since lipophilic Hg complex such as HgCl_2_^0^ could passively diffuse through the biological membranes^51, 52^, OA may decrease the uptake of Hg into the cells.

OA alone had small impacts on the seven life history traits in the copepod *T. japonicus*, in agreement with the previous studies on many copepod species (e.g., *Acartia tsuensis*, *Calanus finmarchicus*, *Calanus glacialis*, *Calanus hyperboreus*, *Centropages typicus, Temora longicornis* and *T. japonicus*) under comparable pCO_2_ concentrations, although most of these earlier studies partially focused on single generation effects by OA^53–57^. Full life cycle tests on *T. japonicus* illustrated that growth rate and hatching success were not affected at 5800 µatm of pCO_2_ (pH~7.11)^57^. Such high resilience of this copepod to elevated pCO_2_ may be explained by their adaptability to their habitats such as tide pool and sea bottom where the pCO_2_ concentration often becomes high. Alternatively, excess food provision could potentially offset the negative impacts of elevated pCO_2_ level on *T. japonicus* in our study, since the copepods might increase their total energy input via compensatory feeding to reallocate the same amount of energy into development and reproduction^58^. However, several other studies showed that OA could strikingly impact the important life traits (e.g., survival rate, egg yielding, and naupliar production) in growth and reproduction of the copepods^59–61^. For instance, Zhang *et al*. investigated the impacts of different elevated pCO_2_ concentrations (800, 2000, 5000, and 10000 μatm) on the survival and reproduction of female *Acartia pacifica*, *Acartia spinicauda*, *Calanus sinicus* and *Centropages tenuiresis* ^59^. They reported that the survival rates and egg hatching success were strongly inhibited by the elevated pCO_2_ with a species-specific manner. Fitzer *et al*. utilized a multigenerational modeling approach to predict a gradual decline in naupliar production of the copepod *Tisbe battagliai* over the next 100 years (equivalent to approximately 2430 generations)^61^. Overall, these works suggested that the responses of copepods to OA were variable and species-specific^53, 57, 59, 61^.

Single Hg exposure only led to the reduced number of nauplii per clutch among the seven life traits examined in the copepod *T. japonicus*. The restrained reproductive performance can be evidenced by our earlier proteomic work that Hg multigenerational toxicity prohibited several critical processes/pathways including vitellogenesis in *T. japonicus* ^22^, since vitellogenesis provides the major egg yolk proteins as essential nutrients for reproduction and early development in oviparous vertebrates and invertebrates. Hook and Fisher also observed a decreased egg production in *Acartia tonsa* and *Acartia hudsonica* following exposure to dissolved Hg concentrations of more than 0.05 µg/L, but this work focused on the single generation exposure^38^. Consequently, Hg pollution suppressed fecundity of the copepods (i.e., population recruitment) and probably affected their community structure and function in marine ecosystem. Cardoso *et al*. reported that the most contaminated areas in a temperate coastal lagoon presented the highest Hg accumulation in zooplankton assemblages with the lowest values of species richness, evenness and heterogeneity^42^. In our study, the inhibitory effects of Hg on number of nauplii per clutch was more obvious in the late generations (i.e., F2-F3), likely ascribed to an increased Hg accumulation in the copepod with generations.

By comparison with the single Hg exposure, number of nauplii/clutch and the fecundity/12 d of the copepods were significantly enhanced by the combined OA + Hg exposure. These were coupled by the decreased development times from nauplius to copepodite and from nauplius to adult (Table 1; Student’s t-test). Clearly, there was an antagonism for OA against Hg toxicity upon developmental time and fecundity in the copepod under multigenerational exposure. Accordingly, OA reduced Hg toxicity, at least in the reproductive performance of the copepod. Again, one likely mechanism was that the reduced Hg accumulation in each generation was observed under the OA + Hg exposure than Hg treatment alone. In contrast to our present work, several previous studies demonstrated a strong synergistic interaction of OA and metal (e.g., Cd and Cu) biotoxicity in marine animals^24, 30^. For example, Campbell *at al.* examined the effects of OA (pCO_2_ 1400 and 3000 µatm corresponding with pH values of 7.77 and 7.47, respectively) on Cu toxicity in the early life history stages of the polychaete *Arenicola marina* and found that the Cu toxicity responses such as sperm DNA damage and early larval survivorship were synergistically enhanced by OA conditions^24^. Meanwhile, a recent study showed that the realistic future ocean pCO_2_ levels (i.e., equal pH values of 7.8 and 7.4) could significantly increase Cd accumulation in the gills, mantle and adductor muscles of three marine bivalves, *Mytilus edulis*, *Tegillarca granosa*, and *Meretrix meretrix* ^26^. Correspondingly, OA and Cd interacted synergistically^30^, even though the free Cd ion concentration may decrease or be unaffected by reduced pH due to OA^23, 28^.

In this work, we showed that near-future OA decreased the Hg bioaccumulation in marine copepod *T. japonicus* under multigenerational exposure. Such reduced accumulation was responsible for the reduced Hg inhibitory effect to the number of nauplii per clutch and total fecundity. These data suggested that OA alleviated Hg toxicity to reproductive performance in marine copepods. Our results were in strong contrast to the synergistic interaction of OA and other metals (e.g., Cd, and Cu) in marine animals. The impact of OA on metal toxicity to marine animals appeared to be metal-specific, which can be explained by the shift of metal speciation, changes in cell surface binding, changes in cell membrane permeability, among others. The mitigation of Hg toxicity in marine copepods under OA scenario was primarily attributed to the reduced metal accumulation as a result of metal-proton competition at the binding sites and lower membrane permeability due to increased H^+^ concentrations.

## Methods

### Copepod maintenance

Copepods *T. japonicus* were obtained from the rocky pools of intertidal zone in Xiamen Bay (People’s Republic of China). They were kept at 22 °C with a 12: 12 h light: dark cycle, and fed on an equal mixture of three algae, *Isochrysis galbana*, *Platymonas subcordiformis*, and *Thalassiosira pseudonana* with a density of 8 × 10^5^ cells/L.

### Seawater chemistry

The seawater was obtained 20 km offshore in Xiamen Bay and was filtered through a 0.22 μm polycarbonate membrane. The background value for T-Hg concentration in the seawater was 3–4 ng/L, the ambient seawater pH was 8.10, and the other seawater parameters are described in Table 2.

**Table 2:** Seawater parameters for experimental treatments.

pCO_2_ (µatm)	Hg levels (µg/L)	Salinity (PSU)	pH				Total alkalinity (µmol/Kg)			
			F0	F1	F2	F3	F0	F1	F2	F3
400	control	27–28	8.045 ± 0.0597	8.033 ± 0.0756	8.014 ± 0.0737	7.989 ± 0.0734	2019.0 ± 94.7	2034.9 ± 121.1	1878.6 ± 117.5	1991.1 ± 52.0
	1.0	27–28	8.049 ± 0.0651	8.038 ± 0.0721	8.011 ± 0.0576	8.004 ± 0.0809	1993.2 ± 120.2	2085.2 ± 4.4	1956.3 ± 50.1	2013.0 ± 17.7
1000	control	27–28	7.643 ± 0.0490	7.694 ± 0.0685	7.685 ± 0.0721	7.653 ± 0.0560	1950.6 ± 107.6	2027.1 ± 131.6	1862.0 ± 157.7	1967.4 ± 67.9
	1.0	27–28	7.643 ± 0.0509	7.680 ± 0.0668	7.685 ± 0.0657	7.643 ± 0.0527	1803.9 ± 100.0	2061.2 ± 19.9	1904.9 ± 55.8	2017.5 ± 27.4

Different pCO_2_ (400, 1000 μatm) and Hg (no Hg addition as control, and 1.0 µg/L) treatments (alone or combined) were utilized in the multigenerational exposure. A total of four treatments were designated, including pCO_2_ 400 μatm + control (specifically regarded as ambient condition), pCO_2_ 1000 μatm + control, pCO_2_ 400 μatm + 1.0 µg/L Hg, and pCO_2_ 1000 μatm + 1.0 µg/L Hg, respectively. The pCO_2_ levels of 400 and 1000 μatm were chosen to represent the present-day condition and the near-future level for the ocean scenario in the year 2100, respectively^62^. The used Hg concentration (1.0 µg/L) was quite high, but still environmentally relevant^19, 20^. The desired pCO_2_ levels were achieved by continuous bubbling with the ambient air or CO_2_-enriched air into filtered seawater in 250 mL polycarbonate bottles. The CO_2_-enriched air was prepared by mixing air and pure CO_2_ using a CO_2_ enrichment device (Ruihua, China). Therefore, the pH values in the present-day (400 μatm) and acidified seawater (1000 μatm) were approximately 8.10 and 7.70, separately (Table 2). The final nominal Hg concentration of 1.0 µg/L was obtained by adding HgCl_2_ (Sigma-Aldrich, 99.5%) into seawater.

### Multigenerational experiments

The multigenerational experiment was carried out in an incubator at 22 °C and 12: 12 h of light and dark cycle. Fifty newly-hatched nauplii (<24 h) were added into polycarbonate bottles with 150 mL seawater in three replicates (total 150 nauplii). These nauplii were maintained under OA and Hg stress (alone or combined) until adult females developed egg sacs. Exposure solutions were daily renewed (~80% of the working volume) with filtered, pCO_2_ and Hg concentration-adjusted seawater, and the alga *P. subcordiformis* was provided as food at a density of approximately 6 × 10^5^ cells/L. In total, seven life history traits, i.e., survival rate, sex ratio (F/M), developmental time from nauplius to copepodite, developmental time from nauplius to adult, number of clutches, number of nauplii/clutch and fecundity were quantified in this study. These parameters were examined for each individual copepod, as described earlier^35^. In brief, developmental stages were observed daily under a stereomicroscope and recorded to calculate the time of development from nauplius to copepodite and from nauplius to adult with egg sacs (i.e., maturation). The development of the egg sac was regarded as the time for maturation. The survival (percentage) and sex ratio were determined after the maturation of all copepods. To measure fecundity, depicted as the number of clutches, and number of nauplii/clutch, six females bearing an egg sac per treatment were individually reared in a new six-well plate with 8 mL of working solution. These females were kept under the above-depicted conditions for 12 d. The resulting nauplii and unhatched clutches were counted and removed under the stereomicroscope. During 12 days, all the six-well plates were placed in two tight boxes, where the pCO_2_ levels were maintained by a continuous supply of the ambient air or CO_2_-enriched air as above-designed.

For F1 (the second generation), fifty nauplii (F1) produced by the first or second brood from each F0 female were maintained in 250 mL polycarbonate bottles (150 mL seawater). The experimental procedure had the same exposure conditions as those used for the F0 testing. The copepods from the subsequent generations were treated as the same conditions as for those in F0, and this long term exposure was kept until the nauplii (F3) developed to maturation. All the adult copepods surviving after the exposure per generation were collected for analyzing T-Hg accumulation.

Seawater parameters such as temperature, salinity, pH, and total alkalinity (TA), were recorded and adjusted as needed in each generation during the exposure. The pH in the exposure solution was daily detected using a pH meter (Thermo Scientific, USA). Exposure seawater samples were collected three times for each generation, and filtered through 0.45 μm membranes (to remove impurities, algae and slough of copepods in seawater) to determine TA by automated spectrophotometric analyzer based on single-point titration and spectrophotometric pH detection^63^.

### T-Hg concentration analysis

To analyze T-Hg contents in the adult copepods of F0-F3, approximately 50 adult copepods were collected and pooled together as a sample with three replicates per treatment. After freeze-drying for 2 days, the samples were digested in a water bath (95 °C) using concentrated HCl and HNO_3_ (1:3, v/v) before testing^64^. T-Hg concentrations in the digestion were measured via a DMA-80 direct mercury analyzer (Milestone, Italy, referred to EPA Method 7473). The minimum detection level for T-Hg is 0.2 ng/g. Mercury standard solutions were analyzed for T-Hg in each batch of samples, and the recovery rates were 85–110%^64^. T-Hg contents in the adult copepods were measured as ng/g dry weight (DW). Additionally, the DCF was calculated as the T-Hg contents in the copepods divided by the nominal metal concentration in the seawater for F0-F3.

### Statistical analysis

All experiments were replicated three times (n = 3), and all the data were presented as mean values ± standard deviation. All the statistical analysis was performed using the software SPSS 19.0. One-way ANOVA and the Fisher least significant difference test were used to evaluate whether the means were significantly different among the groups. Significant differences were indicated at *p* < 0.05. Prior to one-way ANOVA, data were log transformed to meet ANOVA assumptions of normality and variance homoscedasticity.

Also, a student’s t-test of two independent-samples (*p* < 0.05) was performed to determine whether the combined effect of OA plus Hg pollution was significantly different from the single Hg treatment on developmental time from nauplius to copepodite, developmental time from nauplius to adult, number of nauplii/clutch and fecundity under multigenerational exposure.
